# The relationship between protein sequences and their gene ontology functions

**DOI:** 10.1186/1471-2105-7-S4-S11

**Published:** 2006-12-12

**Authors:** Zhong-Hui Duan, Brent Hughes, Lothar Reichel, Dianne M Perez, Ting Shi

**Affiliations:** 1 Department of Computer Science, University of Akron, Akron, OH, 44325, USA; 2 Department of Mathematical Sciences, Kent State University, Kent, OH, 44242, USA; 3 Department of Molecular Cardiology, Lerner Research Institute, Cleveland Clinic Foundation, Cleveland, OH, 44195, USA

## Abstract

**Background:**

One main research challenge in the post-genomic era is to understand the relationship between protein sequences and their biological functions. In recent years, several automated annotation systems have been developed for the functional assignment of uncharacterized proteins. The underlying assumption of these systems is that similar sequences imply similar biological functions. However, it has been noted that matching sequences do not always infer similar functions.

**Results:**

In this paper, we present the correlation between protein sequences and protein functions for the yeast proteome in the context of gene ontology. A novel measure is introduced to define the overall similarity between two protein sequences. The effects of the level as well as the size of a gene ontology group on the degree of similarity were studied. The similarity distributions at different levels of gene ontology trees are presented. To evaluate the theoretical prediction power of similar sequences, we computed the posterior probability of correct predictions.

**Conclusion:**

The results indicate that protein pairs of similar biological functions tend to have higher sequence similarity, although the similarity distribution in each functional group is heterogeneous and varies from group to group. We conclude that sequence similarity can serve as a key measure in protein function prediction. However, the resulting annotations must be verified through other means. A method that combines a broader range of measures is more likely to provide more accurate prediction. Our study indicates that the posterior probability of a correct prediction could serve as one of the key measures.

## Background

The human genome project and numerous other genome projects have produced a large and ever increasing amount of sequence data. One of the main research challenges in the post-genomic era is to understand the relationship between the nucleotide sequences of genes and the functions of the proteins they encode. Traditionally, the functional annotation of genes has been done manually by experienced individual curators with the help of advanced searching tools. However, to unlock the potential of the huge amount of genomic-wide sequence data, it is necessary to develop large-scale approaches for the functional assignment of uncharacterized proteins [1-10]. In recent years, several automated annotation systems have been developed based on homologues identified from database searches, text mining, gene ontologies, and co-expression relationships obtained from microarray gene expression patterns [11-23]. In sequence similarity-based approaches, the function of a query protein can be deduced from those of homologous proteins of known functions obtained from database searches. The underlying assumption of these approaches is that similar sequences imply similar biological functions. Since this assumption is true in many cases and the approaches are simple, this type of sequence matching schemes have been most popular and widely used, although it has been noted that matching sequences do not always infer similar functions [24-26].

The Gene Ontology (GO) consortium provides a vocabulary to describe gene and gene product attributes in any organism [27]. GO includes three ontological categories: molecular function, biological process, and cellular component. A molecular function GO term represents a biological activity involving one or more gene products. A biological process GO term represents a series of biological activities. And a cellular component GO term, as the name suggests, represents a component of a cell. The GO terms in each category are organized in a directed acyclic graph (DAG), i.e., a specialized GO term (child) could be associated with one or several less specialized GO terms (parents).

Since the establishment of GO, many ontology-based sequence annotation approaches have been developed [16-23], including several web-based automated GO annotation software tools [18,19]. These attempts typically involve a search of homologous proteins in GO-mapped databases including Genbank and Swiss-Prot. Hennig et al.'s OntoBlast and Zehetner's GOB let present a list of homologues together with their GO terms [18,19]. Martin et al.'s GOtcha searches a set of seven model genomes and returns scored matches [20]. Xie et al.'s GO Engine combines homology search with text mining [17]. Schug et al. developed a rule-based function prediction method based on the intersection of GO terms that contain protein domain at different similarity levels [16]. Abascal et al. presented an automatic annotation method based on protein family identification [21]. Jensen et al. used neural networks for the prediction while Vinayagam et al. used support vector machines [22,23]. The appeal of these approaches is that they can directly assign a biological meaning to an uncharacterized protein sequence.

In this study, we investigate the mathematical underpinnings of the automated sequence annotation approaches that are based on sequence similarity and gene ontology. We explore the structures of the three ontology categories and re-evaluate the assumption that similar sequences give rise to similar biological functions. We introduce a novel measure of overall similarity between two protein sequences based on a set of local BLAST alignments. Using the complete proteome from the model organism yeast, we study the degree of overall similarity of yeast protein sequences in each functional group defined by GO terms. We examine the effects of the level of GO terms and the size of GO groups on the degree of similarity. We present the sequence similarity distributions at different levels of GO DAGs and the distributions of siblings of GO groups. To evaluate the theoretical prediction power of similar sequences, we compute the posterior probability of the hypothesis that protein A possesses the same biological function as protein B, given B's biological function is known and A and B are similar.

## Results and Discussion

All-to-all pair-wise protein sequence local alignments were performed using the alignment tool for blasting two sequences (I) which was retrieved from the NCBI ftp site [28]. The *p*-values were calculated based on a novel measure (Equation (2) in Methods section) of overall similarity of two protein sequences. The distributions of the *p*-values are shown in Table 1. The first column presents the *p*-value distribution of protein sequence pairs from the complete yeast proteome. This distribution serves as a control for the distribution of the whole population. The second, third and fourth columns show the distributions for sequences annotated for biological processes, molecular functions and cellular components, respectively. As we can see, the four distributions are quite similar, indicating that the annotated proteins in each of the three gene ontology categories provide a representative sample set of sequences from the complete yeast proteome. On the other hand, we clearly see that the majority of sequence pairs are not similar. Only about 4% of the sequence pairs have *p*-values less than 0.01.

**Figure 1:**
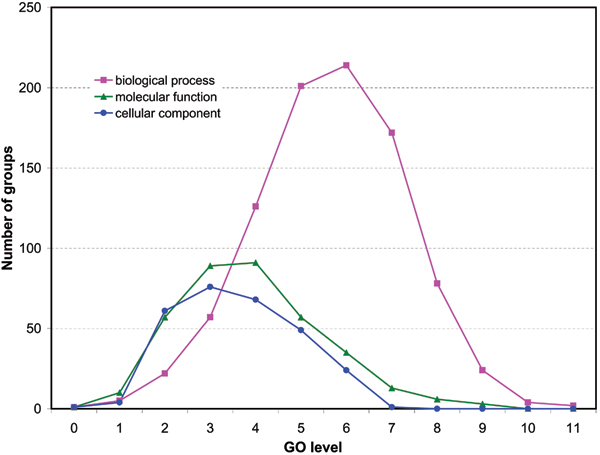
Numbers of GO groups at different levels of the gene ontologies.

**Figure 2:**
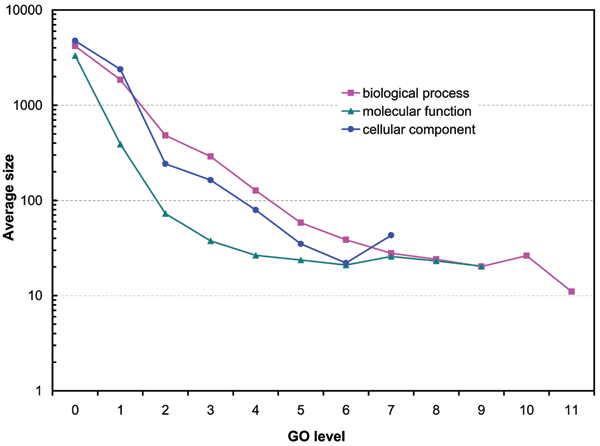
Average size of GO groups at different levels of the ontologies.

The distributions of the number of GO groups at different levels of the gene ontologies are shown in Figure 1. In this study, when a pair of sequences appears on multiple levels, the highest level (most specialized level) was chosen for the analysis. We see clearly that the GO groups in molecular function and cellular component populate the third and the fourth level of the ontologies while the biological process GO groups are mainly distributed around level six. The average sizes of GO groups at different levels of the ontologies are shown in Figure 2. We see that in all three GO categories the average size of the GO groups decreases in most of the cases as their level increases. We note that groups of less than six protein sequences are not shown.

**Figure 3:**
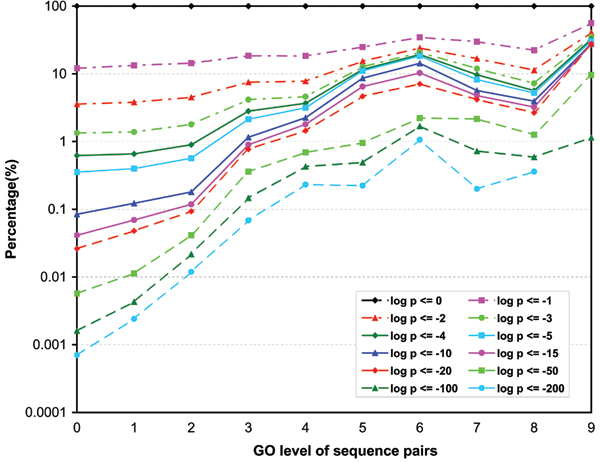
The p-value distributions of sequence pairs annotated for molecular function. Each curve represents the percentages of sequence pairs of less than or equal to certain p-value across different levels of the GO category. (Some curves do not include the percentages for all levels because no sequence pair on those levels has a p-value less than or equal to certain thresholds.)

**Figure 4:**
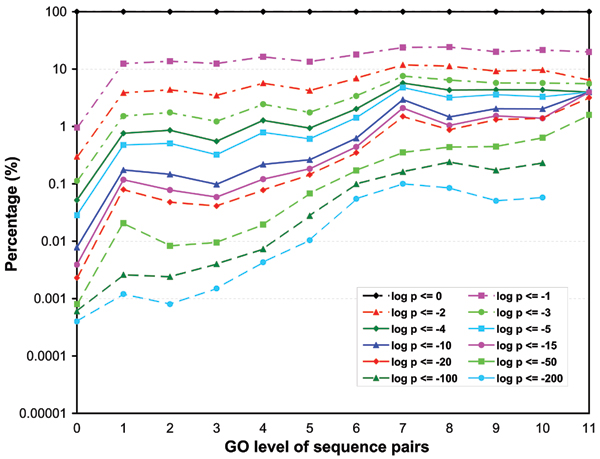
The p-value distributions of sequence pairs annotated for biological process. Each curve represents the percentages of sequence pairs of less than or equal to certain p-value across different levels of the GO category. (Some curves do not include the percentages for all levels because no sequence pair on those levels has a p-value less than or equal to certain thresholds.)

**Figure 5:**
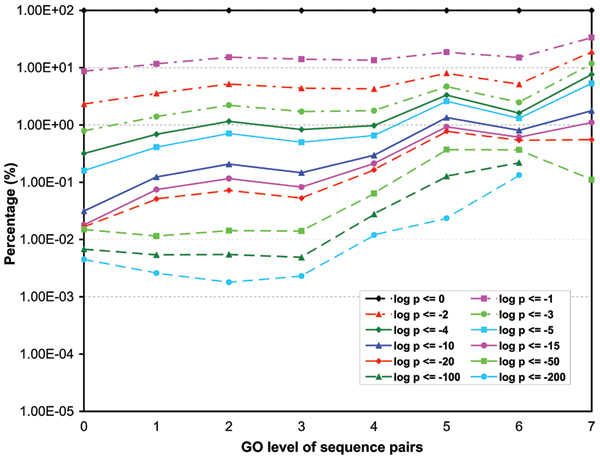
The p-value distributions of sequence pairs annotated for cellular component. Each curve represents the percentages of sequence pairs of less than or equal to certain p-value across different levels of the GO category. (Some curves do not include the percentages for all levels because no sequence pair on those levels has a p-value less than or equal to certain thresholds.)

Figures 3, 4, 5 show the *p*-value distributions of protein sequence pairs annotated for molecular function, biological process and cellular component GO terms at different levels of GO categories. Each curve in the figures represents the percentages of sequence pairs of less than or equal to a certain *p*-value across different levels of GO categories. Some curves do not include the percentages for all levels because no sequence pair on those levels has a *p*-value less than or equal to certain thresholds. For the sequences annotated for molecular function and cellular component GO terms, we see clearly that the majority of the sequence pairs are considered non-similar throughout the levels. Over 59% of sequence pairs at all levels have *p*-values greater than 0.01. However, the number of similar sequence pairs does increase steadily with their GO levels. In particular, the percentage of pairs with high similarity scores (*p *≤ 10^-10^) has a steep increase from the root level to level 5. Level 9 has the highest percentage of similar pairs for molecular function ontology. At this level over 35% of the sequence pairs have similarity *p*-values less or equal to 10^-3 ^while for levels 5 through 8, over 13% of the sequence pairs have *p*-values less or equal to 10^-3^. These percentages are significantly higher than the 1.8% extracted from the *p*-value distribution of the entire population of sequence pairs annotated for molecular functions (Table 1). Also we can see that the percentage increase is not monotonic from levels 6 to 9. There is a short trend that the percentage decreases with level. We believe that this result is mainly due to the nature of the ontology graph in which fewer GO terms are on levels higher than 6. Another reason that may also possibly contribute to the result is that in our analysis, the level of a GO term is defined to be the lowest level on which it appears in the GO DAG. For the cellular component ontology, level 7 has about 12% of the sequence pairs with *p*-values less than or equal to 10^-3^. The average for levels 5 and 6 is about 3.6%. We also see that for the two ontologies, significantly more pairs have high similarity scores (*p *≥ 10^-10^) at levels 5 or above than those at levels below 5. For the biological process ontology, the increase of the number of similar pairs starts to level off around level 7, apparently much higher than for molecular function and cellular component ontologies. About 6.2% of pairs at levels 7 through 11 have the similarity *p*-values less than and equal to 10^-3^, compared to an average of 1.74% at levels below 7. Similar to the two other ontologies, there are significantly more pairs annotated for biology process GO terms having high similarity scores (*p *≥ 10^-10^) at levels 7 and above than at levels below 7.

The complete *p*-value distributions of sequence pairs for each GO group of the three ontologies are shown in the supplement tables I, II, III (Additional data file 1, 2, 3). Table 2 shows a typical part of the supplement table II. It presents the *p*-value distribution of sequence pairs in some GO groups on the transporter activity branch of the molecular function ontology tree. Numbers in each row of the table represent the percentages of sequence pairs of *p*-values within certain range. We see very much diversified *p*-value distributions over different GO groups. Most of the distributions are independent of the sizes of the groups. More noticeably, the sequence pairs in the carbohydrate transporter activity group have much higher similarity scores. Over 75% of pairs have the *p*-values less than or equal to 10^-5^. In particular, the 17 sequences in the sub-group monosaccharide transporter activity are extremely similar with each other. All the *p*-values of the 136 pairs are less than or equal to 10^-50^. On the other hand, the sequences in the GO group monovalent inorganic cation transporter activity which is at the same level as monosaccharide transporter activity exhibits much low similarity scores. More than 96% sequence pairs have *p*-values greater than 10^-2^. Also we see, in general, within one branch of the ontology tree, the higher level a GO group is at, the higher similarity its sequence pairs have. More convincingly, 707 out of 903 biological process groups, 304 out of 362 molecular function groups, and 216 out of 284 cellular component groups have higher percentage of sequence pairs of *p*-values less 10^-3 ^than those of their parents (in the case of multiple parents, the averages of similarity scores of the parents are considered). This result indicates the strong correlation between sequence similarity and function similarity/specificity.

The dependence of sequence similarity on group size was also examined. No strong correlation was found although there is a vague trend of increasing degree of sequence similarity as the group gets smaller. The Pearson correlation coefficient of group size versus percentage of sequence pairs with *p*-values less than or equal to 10^-5 ^for molecular function ontology is about -0.124. The coefficients for biological process and cellular component are -0.137 and -0.136, respectively. As an example, the protein kinase activity group has 94 annotated sequences. About 70% of the 4371 sequence pairs have *p*-values less than or equal to 10^-5^. On the other hand, the nucleobase, nucleoside, nucleotide kinase activity group has only 10 annotated sequences. Only 10 out of the 45 pairs have *p*-values less than or equal to 10^-5^, although both groups are at the same level (level 5) of the molecular function ontology tree. The fact that most child groups have higher similarity scores than their parents might be the main factor contributing to the weak negative correlation between sizes and similarity scores.

The above results indicate that proteins of similar biological functions tend to have higher sequence similarity. The level of GO groups on a gene ontology tree depicts to a certain degree the functional similarity of the groups, although it's far from being able to accurately characterize the relationship between protein sequence similarity and biological function similarity. To evaluate how much protein sequence similarity can contribute to biological function prediction, we computed the posterior probabilities of correct predictions using equation (4). The results for the protein kinase activity branch of the molecular function ontology tree are presented in Table 3 while the *p*-value distributions of sequence pairs for the branch are shown in Table 4 for comparison. As we can see from the results, the posterior probability of a correct assignment varies greatly from group to group. For example, if a database search hits the nucleotide kinase activity group with a *p-*value less than or equal to 10^-100^, one can almost be certain that the protein with that query sequence belongs to the nucleotide kinase activity group. On the other hand a hit to the protein kinase activity group with the same *p*-value would carry only 13% of the confidence that the protein belongs to the group. We believe that the high degree of variation observed in the posterior probabilities indicate that the posterior probability could serve as a key measure in protein function predictions.

## Conclusion

In this paper, we studied the correlation between protein sequence similarity and function similarity for the yeast proteome in the context of the three gene ontologies. The results indicate that protein pairs in a GO group tend to have higher sequence similarity than a randomly drawn sequence pair, although the *p*-value distributions of sequence pairs in GO groups are heterogeneous and vary from group to group. We conclude that sequence similarity can serve as one of the key measures in protein function prediction. However, the results do not directly translate into a high confidence of the function prediction provided by automated protein annotation systems that are solely based on sequence similarity and GO definitions. These methods can serve as a preliminary tool for functional predictions. The resulting annotations have to be verified through other means. A method that combines a broader range of measures, including sequence similarity, GO definitions, gene expression patterns, as well as available knowledge of the organism under study, is more likely to provide more accurate function prediction. Our study indicates that the posterior probability of a correct prediction could serve as one of those key measures.

## Methods

The complete yeast (*Sacchyromyces cerevisiae*) proteome was obtained from Swiss-Prot [29] on July 2005. It includes 6467 protein sequences. GO definition files were obtained from the Gene Ontology consortium web site [30]. In the version of July 1^st ^of 2005, there are 19094 GO terms including 9856 biological process terms, 7559 molecular function terms, and 1679 cellular component terms. Among the 6467 protein sequences, 4175 are annotated with 1084 biological process terms, 3317 are annotated with 1060 molecular function terms, and 4735 are annotated with 354 cellular component terms.

Sequence similarities can be measured through pair-wise global alignments or local alignments. Homologous protein sequences are usually similar over active domains and thus share common folds and functions. Therefore, local alignment is a more appropriate method for comparing protein sequences for their functional similarity. There are several local alignment schemes for comparing protein sequences, including BLAST that can be used together with different scoring systems such as BLOSUM62 and BLOSUM80. The program returns a list of local alignments of certain statistical significance. However, how to measure the overall similarity of two protein sequences is not obvious. For example, proteins with two similar domains with certain similarity scores could be considered to be much more similar than proteins with only one domain with a higher similarity score. In this study, we introduce a novel measure of overall similarity of two protein sequences. We utilize the alignment tool for blasting two sequences to obtain the list of optimal local alignments. Let {S_1_,...,S_n_} be scores of a list of best local alignments with certain statistical significance. Instead of using the highest score (max{S_1_,...,S_n_}) in the list to measure the overall similarity of the two protein sequences, we use the following score S to measure the overall similarity of two sequences:



where  stands for the probability of finding a high-scoring segment pair (HSP) with a local alignment score of at least *S*_*i*_, and *E*_*i *_is the expected number of HSPs of score at least *S*_*i *_and can be obtained directly from the alignment tool. Assuming the HSPs are independent of each other, the *p*-value:



measures the probability of finding a pair of protein sequences with a list of scores at least {*S*_1_, ..., *S*_n_}. The corresponding *E*-value for the overall similarity (the expected number of the lists that have scores at least {*S*_1_, ..., *S*_n_}) therefore can be written as:

*E *= -ln(1 - *e*^-*S*^).     (3)

We use the *p*-values and the *E*-values to measure the overall similarity of a pair of protein sequences. Since when |*x*| < 1, -ln(1 - *x*) = *x *+ *x*^2^/2 + *O*(*x*^3^), the *E- *and *p*-values are essentially the same when they are small. For example, when *p *= 10^-5^, |*p *- *E*| is of order 10^-10^. For convenience, we use *p*-values to present our results in this paper. The alignment tool used for blasting two sequences (b12seq) was retrieved from the NCBI ftp site [28]. We used version 2.2.11 with default parameters and the substitution matrix BLOSUM62. All processing scripts were written in Perl.

GO terms in each of the three ontology categories were parsed and stored in a tree structure similar to the one used in AmiGO [31] to form gene ontology trees. Since GO terms are originally organized in a DAG, a GO term may have several parent terms. In this case, the child term appears multiple times on the same or different levels of the tree. In this paper, we define the level of a GO term to be the lowest level on which it appears, i.e. the shortest distance of the GO term from the root. Protein sequences were then parsed and mapped onto the gene ontology trees to form GO groups. GO groups with less than six protein sequences were removed for statistically meaningful results. As a result, 4175 sequences in 906 distinct biological process groups, 3317 in 362 distinct molecular function groups, and 4735 in 284 distinct cellular component groups are included in the analysis. The final biological process ontology tree consists of 11 levels and 11091 tree nodes (GO groups), of which 903 are unique. The molecular function ontology tree consists of 9 levels and 471 tree nodes, of which 362 are unique. The cellular component tree has 7 levels and 1692 tree notes, of which 284 are unique.

To evaluate how much protein sequence similarity can contribute to biological function prediction, the posterior probabilities of correct predictions can be computed using Bayes' theorem [32]:



where G represents a GO group, *ε *represents the *p*-value threshold for a sequence pair *s*_1 _and *s*_2_, while the *p*-value *p*(*s*_1_, *s*_2_) is calculated based on equation (2).

## Authors' contributions

ZHD proposed the research idea, designed and implemented the research approaches, and drafted the manuscript. BH participated in the implementation of the research approaches and the result analysis. LR contributed to the idea of introducing a novel measure for the overall similarity of two protein sequences and participated in the result discussion and the manuscript writing. DMP and TS participated in developing the research idea, provided the explanation of the biological meaning of the results, and contributed to the manuscript writing. All authors read and approved the final manuscript.

## Supplementary Material

Additional data file 1 **The p-value distribution of sequence pairs in GO groups of molecular function ontology**. The p-value distribution of sequence pairs in GO groups of molecular function ontology. This file contains the p-value distribution of sequence pairs in all GO groups of molecular function ontology Click here for file

Additional data file 2 **The p-value distribution of sequence pairs in GO groups of biological process ontology**. The p-value distribution of sequence pairs in GO groups of biological process ontology. This file contains the p-value distribution of sequence pairs in all GO groups of biological process ontology Click here for file

Additional data file 3 **The p-value distribution of sequence pairs in GO groups of cellular component ontology**. The p-value distribution of sequence pairs in GO groups of cellular component ontology. This file contains the p-value distribution of sequence pairs in all GO groups of cellular component ontology Click here for file

## Figures and Tables

**Table 1:** The *p*-value distributions of protein sequence pairs.

*p*-value range	Percentage of pairs	Percentage of pairs in biological process	Percentage of pairs in molecular function	Percentage of pairs in cellular component
[1, 10^-1^)	87.81	86.08	86.92	85.96
[10^-1^, 10^-2^)	8.559	9.466	9.105	9.589
[10^-2^, 10^-3^)	2.244	2.634	2.399	2.676
[10^-3^, 10^-4^)	0.7139	0.8862	0.7671	0.8903
[10^-4^, 10^-5^)	0.2654	0.3493	0.29	0.3485
[10^-5^, 10^-6^)	0.1194	0.1618	0.1342	0.1614
[10^-6^, 10^-10^)	0.1556	0.2208	0.1843	0.2126
[10^-10^, 10^-15^)	0.05141	0.07762	0.06913	0.06826
[10^-15^, 10^-20^)	0.02427	0.03747	0.0342	0.03117
[10^-20^, 10^-50^)	0.03834	0.06107	0.06368	0.04699
[10^-50^, 10^-100^)	0.01032	0.01468	0.016365	0.01087
[10^-100^, 10^-200^)	0.004558	0.005692	0.007037	0.004185
[10^-200^, 10^-300^)	5.74E-05	8.03E-05	7.27E-05	5.35E-05
[10^-300^, 0]	0.004419	0.004935	0.007291	0.002721

**Table 2:** The p-value distribution of sequence pairs in some GO groups on transporter activity branch.

GO ID^1^	*p*-values							GO group description
		
	[1, 10-^2^)	[10^-2^, 10^-5^)	[10^-5^, 10^-10^)	[10^-10^, 10^-20^)	[10^-20^, 10^-50^)	[10^-50^, 10^-100^)	[10^-100^, 0]	
* GO0005215(392)	94.13	3.68	0.66	0.42	0.52	0.26	0.33	transporter activity
** GO0042626(43)	85.6	2.1	2.21	4.1	2.21	1.99	1.77	ATPase activity, coupled to transmembrane movement of substances
*** GO0042625(30)	85.75	1.84	1.61	3.68	3.91	2.07	1.15	ATPase activity, coupled to transmembrane movement of ions
**** GO0019829(19)	92.4	2.92	0	1.75	1.75	0	1.17	cation-transporting ATPase activity
***** GO0046961(18)	92.16	2.61	0	1.96	1.96	0	1.31	hydrogen-transporting ATPase activity, rotational mechanism
**** GO0015662(12)	30.3	0	10.61	19.7	21.21	13.64	4.55	ATPase activity, coupled to transmembrane movement of ions, phosphorylative mechanism
**GO0015267(11)	87.27	3.64	1.82	0	0	1.82	5.45	channel or pore class transporter activity
*** GO0015268(9)	88.89	5.56	2.78	0	0	2.78	0	alpha-type channel activity
**** GO0005216(7)	85.71	4.76	4.76	0	0	4.76	0	ion channel activity
** GO0015238(14)	41.76	10.99	15.38	12.09	10.99	1.1	7.69	drug transporter activity
*** GO0015239(11)	47.27	9.09	14.55	9.09	12.73	0	7.27	multidrug transporter activity
** GO0015144(27)	21.08	1.71	3.13	8.55	23.65	5.7	36.18	carbohydrate transporter activity
*** GO0051119(24)	0.36	1.81	3.62	10.87	30.07	7.25	46.01	sugar transporter activity
**** GO0015145(17)	0	0	0	0	0	11.03	88.97	monosaccharide transporter activity
***** GO0015149(17)	0	0	0	0	0	11.03	88.97	hexose transporter activity
****** GO0005354(6)	0	0	0	0	0	33.33	66.67	galactose transporter activity
****** GO0015578(15)	0	0	0	0	0	0	100	mannose transporter activity
****** GO0005353(15)	0	0	0	0	0	0	100	fructose transporter activity
****** GO0005355(16)	0	0	0	0	0	0	100	glucose transporter activity
** GO0015075(140)	95.6	3.02	0.16	0.29	0.37	0.2	0.36	ion transporter activity
*** GO0046873(38)	91.18	4.98	0.14	0.28	0.71	1.28	1.42	metal ion transporter activity
**** GO0046915(29)	88.67	6.9	0.25	0.25	0.49	1.48	1.97	transition metal ion transporter activity
***** GO0005375(8)	82.14	10.71	0	3.57	0	3.57	0	copper ion transporter activity
***** GO0005381(10)	75.56	6.67	0	0	0	8.89	8.89	iron ion transporter activity
*** GO0042625(30)	85.75	1.84	1.61	3.68	3.91	2.07	1.15	ATPase activity, coupled to transmembrane movement of ions
**** GO0019829(19)	92.4	2.92	0	1.75	1.75	0	1.17	cation-transporting ATPase activity
***** GO0046961(18)	92.16	2.61	0	1.96	1.96	0	1.31	hydrogen-transporting ATPase activity, rotational mechanism
**** GO0015662(12)	30.3	0	10.61	19.7	21.21	13.64	4.55	ATPase activity, coupled to transmembrane movement of ions, phosphorylative mechanism

^1^The tree structure of the transporter activity branch is embedded in the first column of the table. The number of stars before a GO ID indicates the level of the GO group. If a group (A) is at one level higher than the group (B) on a row immediate above, A is a child of B. Otherwise, A is a sibling of the group that is at the same level as A and described on a nearest row above A. The numerical number in the parentheses after the GO ID represents the size of the group.

**Table 3:** Posterior probability of a correct assignment for sequences in GO groups on kinase activity branch.

GO ID	*p*-value threshold							GO group description
		
	1	10^-2^	10^-5^	10^-10^	10^-20^	10^-50^	10–100	
**** GO0016301(161)	1.25	2.70	29.14	30.75	33.48	4.57	18.67	kinase activity
***** GO0019205(10)	0.070	0.21	10.20	29.41	54.53	28.54	99.90	nucleobase, nucleoside, nucleotide kinase activity
****** GO0019201(6)	0.039	0.20	8.11	19.35	60.00	33.33	99.98	nucleotide kinase activity
***** GO0004672(94)	0.72	3.52	30.07	30.37	31.81	33.25	13.76	protein kinase activity
****** GO0004674(65)	0.50	2.70	21.43	20.10	18.71	3.00	14.07	protein serine/threonine kinase activity
******* GO0004693(6)	0.039	0.176	1.21	1.42	2.83	2.86	0	cyclin-dependent protein kinase activity
******* GO0004680(7)	0.046	0.23	1.83	1.34	4.50	6.67	10.00	casein kinase activity
******* GO0004702(8)	0.054	0.33	2.29	2.67	2.64	1.03	5.88	receptor signaling protein serine/threonine kinase activity
****** GO0004713(6)	0.039	0.24	1.70	1.45	0.87	0	0	protein-tyrosine kinase activity
***** GO0019200(15)	0.11	0.23	15.11	25.43	65.24	65.24	28.6	carbohydrate kinase activity
***** GO0001727(8)	0.054	0.16	3.53	4.38	28.58	25.03	25.06	lipid kinase activity
***** GO0004428(12)	0.085	0.15	3.21	4.97	29.39	29.96	33.22	inositol or phosphatidylinositol kinase activity

**Table 4:** The *p*-value distribution of sequence pairs in GO groups on kinase activity branch.

GO ID	*p*-values							GO group description
		
	[1, 10^-2^)	[10^-2^, 10^-5^)	[10^-5^, 10^-10^)	[10^-10^, 10^-20^)	[10^-20^, 10^-50^)	[10^-50^, 10^-100^)	[10^-100^, 0]	
**** GO0016301(161)	70.45	4.28	5.17	10.82	8.48	0.5	0.31	kinase activity
***** GO0019205(10)	66.67	11.11	0	8.89	8.89	0	4.44	nucleobase, nucleoside, nucleotide kinase activity
****** GO0019201(6)	40	20	0	0	26.67	0	13.33	nucleotide kinase activity
***** GO0004672(94)	25.14	5.51	14.37	29.83	23.5	1.14	0.5	protein kinase activity
****** GO0004674(65)	18.89	5.19	17.31	33.65	22.45	1.59	0.91	protein serine/threonine kinase activity
******* GO0004693(6)	33.33	0	0	0	26.67	40	0	cyclin-dependent protein kinase activity
******* GO0004680(7)	47.62	0	19.05	9.52	4.76	14.29	4.76	casein kinase activity
******* GO0004702(8)	0	0	0	42.86	42.86	10.71	3.57	receptor signaling protein serine/threonine kinase activity
****** GO0004713(6)	0	0	26.67	53.33	20	0	0	protein-tyrosine kinase activity
***** GO0019200(15)	78.1	1.9	5.71	0	0	8.57	5.71	carbohydrate kinase activity
***** GO001727(8)	53.57	7.14	14.29	10.71	7.14	3.57	3.57	lipid kinase activity
***** GO0004428(12)	72.73	6.06	6.06	7.58	3.03	1.52	3.03	inositol or phosphatidylinositol kinase activity
